# Impact of storage conditions and premix type on phytase stability

**DOI:** 10.1093/tas/txaa049

**Published:** 2020-04-24

**Authors:** Marut Saensukjaroenphon, Caitlin E Evans, Chad B Paulk, Jordan T Gebhardt, Jason C Woodworth, Charles R Stark, Jon R Bergstrom, Cassandra K Jones

**Affiliations:** 1 Department of Grain Sciences and Industry, College of Agriculture, Kansas State University, Manhattan, KS; 2 Department of Diagnostic Medicine/Pathobiology, College of Veterinary Medicine, Kansas State University, Manhattan, KS; 3 Department of Animal Sciences and Industry, Kansas State University, Manhattan, KS; 4 DSM Nutritional Products, North America, Animal Nutrition and Health, Parsippany, NJ

## Abstract

Potential use of medium-chain fatty acids (MCFA), increased temperatures and exposure time may be implemented to mitigate biological hazards in premixes and feed ingredients. However, there are no data on how these strategies influence phytase stability. For Exp. 1, there were no four- and three-way interactions among premix type (PT), oil type (OT), storage condition (SC), and storage time (ST) for phytase stability (*P* > 0.111). There were two-way interactions for PT × SC (*P* < 0.001) and SC × ST (*P* < 0.001). The OT did not affect phytase stability when premixes-containing phytase were added as either mineral oil (MO) or MCFA (*P* = 0.382). For Exp. 2, there was no interaction between PT and OT (*P* = 0.121). There were also no differences for phytase stability between vitamin premix (VP)- and vitamin trace mineral (VTM) premix-containing phytase were heated at 60 °C (*P* = 0.141) and between premixes-containing phytase were mixed with 1% MO added and 1% MCFA (*P* = 0.957). Therefore, the phytase was stable when mixed with both VP and VTM premix and stored at 22 °C with 28.4% relative humidity (RH). The phytase stability was dramatically decreased when the phytase was mixed with premixes and stored at 39.5 °C with 78.8% RH. Also, MCFA did not influence phytase degradation during storage up to 90 d and in the heat pulse process. The phytase activity was decreased by 20% after the premixes containing the phytase was heated at 60 °C for approximately 9.5 h. If both MCFA and heat pulse treatment have similar efficiency at neutralizing or reducing the target pathogen, the process of chemical treatment could become a more practical practice.

## INTRODUCTION

Phytase is a phosphohydrolytic enzyme that releases phosphorus from phytate in cereal grains, which is a main ingredient in animal feed. This enzyme can be used to reduce phosphorus content in manure by breaking down phosphorus from the phytate to make it available to the animal. Pure phytase products can be added directly in the diet or mixed with vitamin premix (VP) or vitamin trace mineral (VTM) premix to reduce the number of additives being added in the mixer.

Feed ingredients and additives could be a potential medium for bacteria and virus transmission (Jones et al., 2019). Dee et al. (2018) demonstrated that pathogenic viruses, such as porcine epidemic diarrhea virus (PEDV) and African swine fever virus (ASFV), can survive in some feed ingredients and feed additives under simulated transport conditions. Certain feed additives have potential to reduce pathogen contamination. Previous research demonstrated that 1% of a medium-chain fatty acid blend (MCFA) effectively mitigated PEDV in feed ingredients (Cochrane et al., 2016). This leads to potential use of MCFA as a mitigant in premixes and feed ingredients. However, there are no data on the effect of MCFA on phytase stability when it is added in VP- and VTM-containing phytase. Therefore, the first objective of this experiment was to determine the impact of 0-, 30-, 60-, or 90-d storage time on phytase stability when VP- and VTM-containing phytase are blended with 1% inclusion of MCFAs (1:1:1 blend of C6:C8:C10) or mineral oil (MO) with different environmental conditions.

In addition to feed additives, pathogens could be eliminated by a combination between temperature and exposure time. For instance, ASFV can be inactivated at 60 °C for 20 min (OIE, 2009), whereas PEDV activity can be reduced about 5.5 log_10_ when heated at 60 °C for 30 min (Hofmann and Wyler, 1989). Thus, exposing ingredients to increased temperature for a short period of time (a heat pulse treatment) could be an opportunity to prevent pathogen movement from a high-risk area to a clean area. However, one concern is that a heat pulse treatment could denature phytase that is added in the premixes. Therefore, the second objective of this experiment was to determine the effect of heat pulse treatment and MCFA addition on phytase stability with two premix types (PTs).

## MATERIALS AND METHODS

### Mixing Procedure

**Figure 1.**
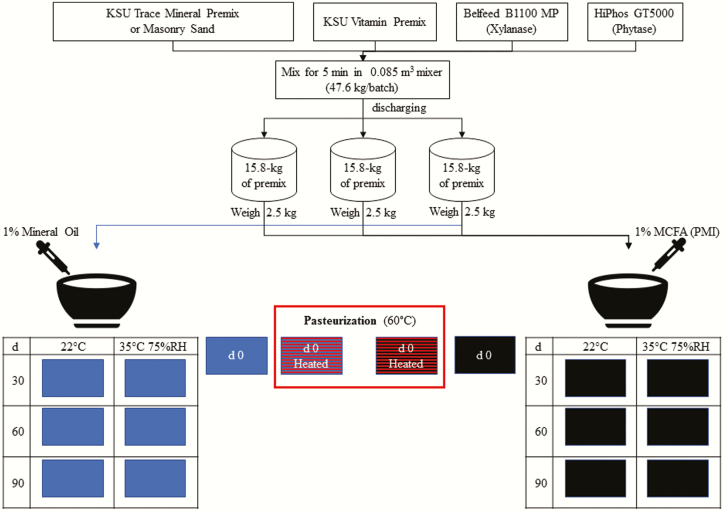
Flow chart of mixing steps used to create experimental treatments. Ingredients were mixed for 5 min in 47.6-kg batches using a 0.085-m^3^ paddle mixer (Davis model 2014197-SS-S1, Bonner Springs, KS). Then, each premix was equally discharged into three separate 15.9-kg aliquots. A 2.5-kg subsample of each aliquot was taken to create a 7.5-kg experimental premix treatment. The 7.5-kg premixes were mixed for 10 s using a mixer (Hobart model HL-200, Troy, OH). Following the 10-s dry mix, either a 74.8 g of 1:1:1 commercial blend of C6:0, C8:0, and C10:0 medium-chain fatty acids (MCFA; PMI Nutritional Additives, Arden Hills, MN) or 74.8-g of mineral oil (MO) was added using a pressurized hand-held sprayer with a fine hollow cone spray nozzle (UNIJET model TN-SS-2, Wheaton, IL). The premixes were mixed for an additional 90 s after oil application. The mixed samples were divided to obtain eight individual 900-g samples, which were placed in single-lined paper bags. Samples were then stored at room temperature in a temperature-controlled laboratory (approximately 22 °C) or in an environmentally controlled chamber (Caron model 6030, Marietta, OH) set at 40 °C and 75% relative humidity (RH). In addition, separate samples were heated in an environmentally control chamber (Caron model 6030, Marietta, OH) at 60 °C and 20% RH.

A VP and VTM premix were manufactured as outlined in Table 1. Ingredients were mixed for 5 min in 47.6-kg batches using a 0.085-m^3^ paddle mixer (Davis model 2014197-SS-S1, Bonner Springs, KS). Then, each premix was equally discharged into three separate 15.9-kg aliquots. A 2.5-kg subsample of each aliquot was taken to create a 7.5-kg experimental premix treatment. The 7.5-kg premixes were mixed for 10 s using a mixer (Hobart model HL-200, Troy, OH) equipped with an aluminum flat beater model HL-20 that had 3.69% coefficient of variation when it was validated for uniform liquid addition. Following the 10-s dry mix, either a 74.8 g of 1:1:1 commercial blend of C6:0, C8:0, and C10:0 MCFA (PMI Nutritional Additives, Arden Hills, MN) or 74.8 g of MO was added using a pressurized hand-held sprayer with a fine hollow cone spray nozzle (UNIJET model TN-SS-2, Wheaton, IL). The premixes were mixed for an additional 90 s after oil application. The mixed samples were divided to obtain eight individual 900-g samples, which were placed in single-lined paper bags. This process was repeated to yield three replicates per treatment. The mixing steps are illustrated in Fig. 1.

**Table 1.** The composition of vitamin premix and vitamin trace mineral premix

	Vitamin trace mineral premix		Vitamin premix	
Ingredients	% Inclusion	Batch, kg	% Inclusion	Batch, kg
KSU swine vitamin^1^	25.89	57.07	54.35	25.89
KSU trace mineral^2^	32.60	15.53	0.00	0.00
Masonry sand	0.00	0.00	32.60	15.53
HiPhos GT5000^3^	8.70	4.14	8.70	4.14
Belfeed B 1100 MP^4^	4.35	2.07	4.35	2.07
Total	100.00	47.63	100.00	47.63

^1^Composition per kilogram: 1,653,000 IU vitamin A; 661,376 IU vitamin D3; 17,637 IU vitamin E; 13.3 mg vitamin B12; 1,323 mg menadione; 3,307 mg riboflavin; 11,023 mg d-pantothenic acid; and 19,841 mg niacin. Rice hulls and calcium carbonate are carriers in the premix.

^2^Composition per kilogram: 73 g iron; 73 g Zinc; 22 g manganese; 11 g copper; 198 g iodine; and 198 g selenium. Calcium carbonate is a carrier in the premix.

^3^Composition per kilogram: 5,000,000 FYT phytase (*Aspergillus oryzae*).

^4^Composition per kilogram: 98,000 U xylanase (*Bacillus subtilis*).

### Experiment 1

The objective of Exp. 1 was to determine the impact of 0-, 30-, 60-, or 90-d storage time on phytase stability when VP- and VTM-containing phytase are blended with 1% inclusion of MCFAs (1:1:1 blend of C6:C8:C10) or MO with different environmental conditions. To achieve this objective, samples were stored at room temperature (RT) in a temperature-controlled laboratory (approximately 22 °C) or in an environmentally controlled chamber (Caron model 6030, Marietta, OH) set at 40 °C and 75% humidity, high heat high humidity. The sample bags were pulled out at days 0, 30, 60, and 90 for RT condition and at days 30, 60, and 90 for high-temperature and high-humidity (HTHH) condition. The actual storage temperature and humidity for both conditions were collected using a data logger (HOBO model Onset U12-012, Bourne, MA). For the RT condition, the average temperature was 22.0, 22.1, and 22.1 °C, and the average relative humidity (RH) was 28.4%, 23.0%, and 33.7% for days 0 to 30, 31 to 60, and 61 to 90, respectively. For the HTHH condition, the average temperature was 39.5, 39.5, and 39.5 °C, and the average RH was 78.3%, 79.0%, and 79.1% for days 0 to 30, 31 to 60, and 61 to 90, respectively. The individual premix samples were riffle divided twice to yield two 200-g subsamples, and then they were sent to laboratories for phytase activity. Phytase activity was analyzed in duplicate according to the official AOAC (2001) method by incubation with sodium phytate. The phytase activity at day 0, which was the initial concentration, was reported in phytase unit FYT per kilogram. The results of phytase at days 30, 60, and 90 were reported in percent stability, which was calculated by dividing the phytase activity by the initial phytase activity and then multiplying by 100.

### Experiment 2

The objective of Exp. 2 was to determine the effect of heat pulse treatment and MCFA addition on phytase stability with two PTs. To achieve this objective, a sample from each treatment was heated in an environmentally control chamber (Caron model 6030, Marietta, OH) at 60 °C and 20% humidity. The sample bags were pulled out after they were stored for 11 h and 48 min. The data logger (HOBO model Onset U12-012, Bourne, MA) was placed within the sample bag at approximately midlevel, and remaining sample was placed on top to ensure data logger reflected true sample temperature. The premix temperature reached 60 °C after 2 h and 21 min in the chamber. The samples were held at 60 °C for 9 h and 27 min. The individual premix samples were riffle divided twice to yield two 200-g subsamples, and then they were sent to commercial laboratories for phytase activity. The results of phytase after heat pulse treatment were reported in percent stability, which was calculated by dividing the phytase activity by the initial phytase activity and then multiplying by 100.

### Statistical Analysis

The initial phytase concentration was analyzed using the GLIMIX procedure of SAS with mixing batch serving as the experimental unit. Treatments were analyzed as a 2 × 2 factorial, with two PTs (VP or VTM) and two oil types (OTs; MO or MCFA). Treatment differences were considered significant at *P* ≤ 0.05.

For Exp. 1, data were analyzed using the GLIMIX procedure of SAS with sample storage bag serving as the experimental unit. Treatments were analyzed as a 2 × 2 × 2 × 4 factorial, with two PTs (VP or VTM), two OTs (MO or MCFA), two storage conditions (RT or HTHH), and three storage times (30, 60, or 90 d). Treatment differences were considered significant at *P* ≤ 0.05.

For Exp. 2, data were analyzed using the GLIMIX procedure of SAS with mixing batch serving as the experimental unit. Treatments were analyzed as a 2 × 2 factorial, with two PTs (VP or VTM) and two OTs (MO or MCFA). Treatment differences were considered significant at *P* ≤ 0.05.

## RESULTS

### Initial Phytase Activity

The initial phytase activity in the VP were 589,162, and 591,144 phytase units (FYT)/kg for MO and MCFA treatments, respectively. Although the initial phytase activity in the VTM premix was 557,478 and 566,410 FYT/kg for MO and MCFA, respectively (Table 2). The formulated phytase activity was 434,884 FYT/kg. The phytase activities of VP with MO, VP with MCFA, VTM with MO, and VTM with MCFA were 1.36, 1.36, 1.28, and 1.30 times greater than the formulated activity, respectively. There was no interaction between PT and OT for the initial phytase activity (*P* = 0.873). The main effects indicated that both PT (*P* = 0.214) and OT (*P* = 0.800) did not affect the initial phytase activity.

**Table 2.** The analyzed phytase activity of initial samples (day 0 and sampled immediately after mixing)

Item		Phytase activity, FYT/kg
Premix type	Oil type^1^	
Vitamin premix	Mineral oil^2^	589,162
Vitamin premix	MCFA^3^	591,144
Vitamin trace mineral premix	Mineral oil	557,478
Vitamin trace mineral premix	MCFA	566,410

^1^Included at 1% of the premixes.

^2^Mineral oil comprised saturated aliphatic and alicyclic nonpolar hydrocarbons sourced as a byproduct of petroleum refining.

^3^Medium chain fatty acid, MCFA, comprised a 1:1:1 blend of medium-chain fatty acids (C6:0, C8:0, and C10:0) (PMI Nutritional Additives, Arden Hills, MN).

### Experiment 1

There were no four- or three-way interactions among combinations of OT, PT, storage condition, and storage time (*P* > 0.111). This experiment indicated there were two two-way interactions between PT and storage condition (*P* < 0.001) and between storage temperature and storage time (*P* < 0.001; Table 3). For the PT × storage condition interaction, the samples stored at HTHH had decreased phytase stability compared with those stored at RT; however, the VTM-containing phytase had greater phytase stability when compared with VP-containing phytase when they were stored at HTHH. There was no difference for phytase stability between VP- and VTM-containing phytase when they were stored at RT. For storage condition × storage time interaction, there was a significant decrease in phytase stability as the storage time increased from 30 to 90 d when the premixes-containing phytase were stored at HTHH, but not when stored at RT. There was no difference for phytase stability when premixes-containing phytase were added either MO or MCFA (*P* = 0.382).

**Table 3.** Effect of the premix type, oil type, storage temperature, and storage time on phytase stability

Item				
Premix type	Storage condition	Storage time, d	Oil type^2^	Phytase stability^1^, %
Interaction				
Vitamin premix	RT^3^			99.8^a^
Vitamin premix	HTHH^4^			15.5^c^
Vitamin trace mineral premix	RT			102.7^a^
Vitamin trace mineral premix	HTHH			29.0^b^
Pooled SEM				1.1
	RT	30		91.4^c^
	RT	60		102.6^b^
	RT	90		109.8^a^
	HTHH	30		43.5^d^
	HTHH	60		15.9^e^
	HTHH	90		7.2^f^
	Pooled SEM			1.3
Main effect				
			Mineral oil^5^	62.2
			MCFA^6^	61.2
			Pooled SEM	1.3
Source of variation				
Oil type				0.382
Premix type				<0.0001
Oil type × premix type				0.641
Storage condition				<0.0001
Oil type × storage condition				0.386
Premix type × storage condition				<0.0001
Oil type × premix type × storage condition				0.280
Time				<0.0001
Oil type × time				0.684
Premix type × time				0.172
Oil type × premix type × time				0.458
Storage condition × time				<0.0001
Oil type × storage condition × time				0.889
Premix type × storage condition × time				0.111
Oil type × premix type × storage condition × time				0.502

^a,b,c,d,e,f^Indicate significant differences (*P* < 0.05) among means within a column.

^1^Percent phytase stability was calculated by dividing the phytase activity at days 30, 60, or 90 by the analyzed initial phytase activity and then multiplying by 100.

^2^Included at 1% of the premixes.

^3^RT = Room temperature, the average temperature, and relative humidity were 22.1 °C and 28.4%, respectively.

^4^HTHH = high heat and high humidity; the average temperature and relative humidity were 39.5 °C and 78.8%, respectively.

^5^Mineral oil comprised saturated aliphatic and alicyclic nonpolar hydrocarbons sourced as a byproduct of petroleum refining.

^6^Medium chain fatty acid (MCFA) comprised a 1:1:1 blend of medium-chain fatty acids (C6:0, C8:0, and C10:0; PMI Nutritional Additives, Arden Hills, MN).

### Experiment 2

There was no interaction between OT and PT (*P* = 0.121). There were also no significant differences for phytase stability between VP- and VTM-containing phytase were heated at 60 °C for 9 h and 27 min (*P* = 0.141) and between premixes-containing phytase were mixed with 1% MO added and 1% MCFA (*P* = 0.957; Table 4).

**Table 4.** Effect of the premix type and oil type on phytase stability of premix subjected to a pulse of high temperature (60 °C)

Item		
Premix type	Oil type^2^	Phytase stability^1^, %
Vitamin premix	Mineral oil^3^	82.0
Vitamin premix	MCFA^4^	78.7
Vitamin trace mineral premix	Mineral oil	81.8
Vitamin trace mineral premix	MCFA	85.4
	Pooled SEM	2.0
Main effect		
Vitamin premix		80.4
Vitamin trace mineral premix		83.6
Pooled SEM		1.4
	Mineral oil	81.9
	MCFA	82.1
	Pooled SEM	1.4
Source of variation		
Oil type × premix type		0.121
Oil type		0.957
Premix type		0.141

^1^Percent phytase stability was calculated by dividing the phytase activity at days 30, 60, or 90 by the analyzed initial phytase activity and then multiplying by 100.

^2^Included at 1% of the premixes.

^3^Mineral oil comprised saturated aliphatic and alicyclic nonpolar hydrocarbons sourced as a byproduct of petroleum refining.

^4^Medium-chain fatty acid (MCFA) comprised a 1:1:1 blend of medium-chain fatty acids (C6:0, C8:0, and C10:0; PMI Nutritional Additives, Arden Hills, MN).

## DISCUSSION

The concern with biological hazards in feed has led to changes in feed manufacturing procedures to ensure feed safety. Therefore, the influence of these procedures on micronutrient and zootechinical feed additive stability (i.e., phytase) is important to determine for a quality assurance. The swine industry first recognized the significant role that the feed supply chain can play in pathogen transfer with PEDV. Since then, it has also been reported that feed was a possible vehicle of Senecavirus A transmission (Leme et al., 2019). Although it is concerning that the domestic spread of viruses has been linked to ingredients, feed, or feed delivery, the larger looming threat is various foreign animal diseases. Such viruses include, foot and mouth disease virus, classical swine fever virus, ASFV, and Chinese pseudorabies virus. Due to the severe consequences of entry, virus transmission through the feed supply chain is a risk worthy of significant investigation and mitigation. Previous data have demonstrated the ability of a virus to spread to surfaces throughout the feed manufacturing facility and stay present until sanitation, which is extremely difficult to properly do in a feed mill (Schumacher et al., 2017). Even with our best efforts to prevent disease entry into a feed mill, there is still the potential for its presence and subsequent transmission through feed. As a final hurdle to prevent transmission to pigs, facilities may consider proactive mitigation through quarantining ingredients, thermal processing, or the use of feed additives. Quarantining ingredients to allow for natural viral degradation may be an effective method of mitigation; however, there is limited information to carry it out successfully. Thermal processing has had demonstrated success to reduce the infectivity of PEDV in feed (Cochrane et al., 2017). The success of thermal process relies on time × temperature combinations that have not yet been fully established. Quarantine time and thermal processing are both considered point-in-time mitigation measures. Both may, under ideal conditions, lead to viral inactivation. However, neither protect the ingredient nor feed from subsequent downstream cross-contamination that may occur during conveyance, load-out, or transportation. Due to the potential for cross-contamination, feed additives may be more successful mitigants. Formaldehyde-based ingredients or those containing MCFA have had demonstrated success as mitigants of porcine pathogens (Schumacher et al., 2017), and their potential is being evaluated in mitigating foreign animal diseases in feed and ingredients. When determining the practical application of these types of ingredients, it is important to consider the ability to use them safely and in compliance with regulatory requirements. In addition, it is important to understand how they influence other nutritional components within the diet.

The results of this experiment indicated that the phytase stability was 99.8% and 102.7% when phytase was mixed with VP and VTM, respectively, after stored at RT, regardless of OT and storage time (up to 90 d). European Food Safety Authority (EFSA, 2012) reported that the phytase stability of Ronozyme Hiphos GT, fine-granular coated form of *Aspergillus oryzae* phytase, when it was added in VP and VTM was 94% and 80%, respectively, after they were stored at 25 °C for 6 mo. The stability of phytase that was added in VP was similar between the present study and EFSA’s report whereas the stability of phytase that was mixed with VTM was higher by 23% in the present study than the EFSA’s report. Moreover, De Jong et al.’s (2016) experiment demonstrated that phytase was less stable when it was mixed with VTM than when it was mixed with VP regardless of storage temperature and phytase source. However, the result of the current study demonstrated that the phytase stability were similar between phytase was mixed in VP and VTM regardless of OT and storage time. Additionally, the phytase stability was 91.4%, 102.6%, and 109.8% when phytase was mixed in premixes and then stored at RT for 30, 60, and 90 d, respectively, regardless of OT and PT. For HTHH, the phytase stability was 15.5% and 29.0% when phytase was added in VP and VTM, respectively, regardless of OT and storage time. The phytase degradation was greater when phytase was added in VP compared to VTM regardless of storage time and OT in contrast to Sulabo et al.’s (2011) study that the phytase degradation was lower when phytase was mixed with VP versus VTM. The reason for different degradation rate between VP and VTM after they were stored under HTHH is unknown. The *A. oryzae* phytase in premixes was degraded overtime when they were stored under HTHH. The phytase stability was 43.5%, 15.9%, and 7.2% when phytase was mixed in premixes then stored under HTHH for 30, 60, and 90 d, respectively, regardless of OT and PT. Jacela et al. (2010) stated that phytase is sensitive to both moisture and temperature when placed in premix which is in agreement with the result of the current experiment. Therefore, the fine-granular coated form of *A. oryzae* phytase was stable in both VP and VTM when stored at RT. However, the phytase in premixes was continuously degraded overtime when stored under HTHH. However, MCFA did not impact phytase stability during storage up to 90 d. This degradation of phytase activity over time when stored under HTHH is important for swine nutritionist to consider when formulating diets. Vier (2019) demonstrated that three different sources of phytase stored in a VTM premix at 30 °C and 75% RH for 90 d resulted in phytase stabilities ranging from 38% to 54%. When these premixes were added to the diet of nursery pigs, feed efficiency and bone mineralization were reduced.

Previous data have demonstrated that heating serum and body fluids to 60 °C for 15 min is sufficient to inactivate ASFV in pig slurry (Turner and Williams, 1999). Therefore, if these procedures were used to reduce potential contamination of ASFV in feed, it is important how they may influence other nutritional components of the diet. In the experiment reported herein, samples were held at 60 °C for 11 h and 48 min. The phytase stability was 80.4% and 83.6% when phytase was added in VP and VTM, respectively, after heated at 60 °C for 11 h and 48 min regardless of OT. Correspondingly, Chantasartrasamee et al. (2005) reported that phytase activity stability of *A. oryzae* phytase was 81% after phytase was heated at 60 °C for 2 h. In addition, the result of the present study demonstrated that the phytase stability was similar between when phytase was added in premixes with 1% MO added and with 1% MCFA added. Therefore, MCFA did not increase the degradation of phytase when it was added in the premixes. Phytase stability was approximately 82% when the premixes were heated at 60 °C for approximately 9.5 h.

## CONCLUSION

The fine-granular coated form of *A. oryzae* phytase was stable when mixed with both VP and VTM premix and stored at 22 °C with 28.4% RH. The phytase stability was dramatically decreased when the phytase was mixed with premixes and stored at 39.5 °C with 78.8% RH. In addition, MCFA did not influence phytase degradation during storage up to 90 d and in the heat pulse process. The phytase activity was decreased by 20% after the premixes containing the phytase was heated at 60 °C for approximately 9.5 h. If both chemical treatment (MCFA) and heat pulse treatment have similar efficiency at neutralizing or reducing the target pathogen, the process of chemical treatment could become a more practical practice.
